# Sex differences in the association of math achievement with visual‐spatial and verbal working memory: Does the type of math test matter?

**DOI:** 10.1111/bjop.12562

**Published:** 2022-03-29

**Authors:** Eva van de Weijer‐Bergsma, Johannes E. H. Van Luit, Korbinian Moeller

**Affiliations:** ^1^ Department of Pedagogical and Educational Sciences Faculty of Social and Behavioral Sciences Utrecht University TC Utrecht The Netherlands; ^2^ Centre for Mathematical Cognition School of Science Loughborough University Loughborough UK; ^3^ Leibniz‐Institut für Wissensmedien Tübingen Germany; ^4^ LEAD Graduate School and Research Network University of Tübingen Tübingen Germany

**Keywords:** math fluency, math problem solving, primary school, sex differences, working memory

## Abstract

Previous research on sex differences in mathematical achievement shows mixed findings, which have been argued to depend on types of math tests used and the type of solution strategies (i.e., verbal versus visual‐spatial) these tests evoke. The current study evaluated sex differences in (a) performance (development) on two types of math tests in primary schools and (b) the predictive value of verbal and visual‐spatial working memory on math achievement. Children (*N* = 3175) from grades 2 through five participated. Visual‐spatial and verbal working memory were assessed using online computerized tasks. Math performance was assessed five times during two school years using a speeded arithmetic test (math fluency) and a word problem test (math problem solving). Results from Multilevel Multigroup Latent Growth Modeling, showed that sex differences in level and growth of math performance were mixed and very small. Sex differences in the predictive value of verbal and visual‐spatial working memory for math performance suggested that boys seemed to rely more on verbal strategies than girls. Explanations focus on cognitive and emotional factors and how these may interact to possibly amplify sex differences as children grow older.

## BACKGROUND

Sex differences in mathematics have been widely investigated in the last decades following initial evidence of a male advantage in mathematical achievement (Geary, 1996; Halpern et al., 2007; Zhu, 2007). Although findings are somewhat mixed, with an increasing number of studies indicating similar mathematical achievement across the sexes (Lachance & Mazzocco, 2006; Lindberg et al., 2010), certain patterns seem to emerge from studies that do find sex differences. Amongst others, these patterns indicated that the type of measure that is used for mathematical achievement seems to affect which sex is in advantage (Halpern et al., 2007). Additionally, these mixed findings are assumed to be related to different solution strategies that different types of math tests may elicit and sex differences in visual‐spatial and verbal processing involved in these solution strategies (Hyde, 2016; Levine et al., 2016).

In the current longitudinal study, we evaluated whether female and male primary school students differed in (the development of) their achievement on two types of mathematical tests, and how this was related to their visual‐spatial and verbal working memory (WM) abilities.

As performance gaps in mathematics may increase from the start of formal education (Gibbs, 2010), suggesting possible sex differences in growth, we focused on mathematical achievement level as well as achievement growth. Such early‐but‐small differences in math achievement may precede more pronounced disparities in math‐related career paths. That is, sex differences in mathematical achievement are seen as an important factor explaining the underrepresentation of women in the most mathematically intensive fields in Science, Technology, Engineering, and Mathematics (Wang & Degol, 2017). The findings of this study should allow for new insights into the developmental pathways of sex differences in mathematical achievement and their potential determinants. As such, identifying cognitive factors that may exacerbate or reduce this gap is vital and can inform curriculum developers and educational professionals how they can develop materials and instruction that reduce sex differences.

### Sex differences in mathematical achievement

Mathematical ability includes a broad set of skills, like number sense, arithmetic fact knowledge, accurate or fluent calculation, and mathematical problem solving or reasoning. Within the broad domain of mathematics, sex differences – when observed – usually favour males over females (Reilly et al., 2015). It should be noted, however, that many studies investigating sex differences in numerical and mathematical skills use data from large‐scale assessments such as PISA (OECD, 2018) or TIMSS (Mullis et al., 2016), which rely mostly on mathematical word problem‐solving tasks.

The PISA data, for instance, showed on average, across the 65 participating countries, that 15‐year‐old boys outperform girls, and that these differences are most pronounced in the highest achieving groups of students (OECD, 2018). The TIMSS data showed similar patterns in 4th and 8th‐grade students, with boys performing better than girls on average (Mullis et al., 2016). Such sex differences favouring males have been observed in primary school (Geary et al., 2000) and seem to increase during secondary school and adulthood (Gallagher et al., 2000; Reilly et al., 2015).

Importantly, however, when differentiating between content strands within mathematics, findings appear to be more mixed. In TIMSS for example, boys performed better on number and geometry tasks, while girls performed better on algebra and data/measurement tasks (Mullis et al., 2016). These mixed findings with regard to sex differences in mathematics thus seem to be related to the type of mathematics measure used (Halpern et al., 2007).

### A verbal versus visual‐spatial ability explanation

One of the explanations for these mixed findings regarding sex differences considers the type of cognitive abilities that certain mathematical tests draw upon and the type of solution strategies females and males tend to use more prominently (Geary et al., 2000; Miller & Halpern, 2014). More specifically, a verbal versus visual‐spatial ability explanation for sex differences in math achievement has been suggested, considering established sex differences in visual‐spatial and verbal abilities (Hyde, 2016; Levine et al., 2016).

In line with evidence indicating a female advantage in verbal or language abilities, such as reading and writing (Halpern et al., 2007; Miller & Halpern, 2014; Zhu, 2007), females were suggested to have an advantage when mathematical problems require more verbal and classroom‐learned solution strategies (Wei et al., 2012; Zhu, 2007). For instance, a female advantage was found for tasks such as exact math problems (e.g., 284 + 369 = …. or 35 × 71 = ….) that primarily rely on procedures taught in the math curriculum, as well as on tasks that rely on the retrieval of basic math facts that are stored verbally in long term memory (although this difference seems to disappear in secondary school years; Halpern et al., 2007). For instance, a study by Wei et al. (2012) in third to 6th grade students showed that girls outperformed boys on exact math tasks, but that these sex differences were eliminated when word‐rhyming scores were controlled for, which substantiated that the female advantage in exact math facts may be (partly) due to advantages in verbal processing.

Moreover, in line with evidence that shows a male advantage in visual‐spatial abilities, such as mental rotation (Halpern et al., 2007; Miller & Halpern, 2014; Zhu, 2007), males were found to have an advantage in mathematical tasks that require spatial strategies, such as mental manipulation of images (Kaufman, 2007). A male advantage in mathematical word problem solving was found already in early primary school and seems to extend into adolescence (Filippetti & Richaud, 2016; Geary et al., 2000). This is often explained by the fact that mathematical (word) problems often favour spatially based solution strategy (Bull et al., 2013; Zhu, 2007).

Consider, for instance, the following word problem: “Trees shall be planted along a street with one tree every 50 m over a length of 600 m starting with a tree at the beginning of the street. How many trees are needed?” (Hegarty & Kozhevnikov, 1999). This word problem suggests simply dividing 600 by 12 to derive the number of trees. However, it needs to be considered that there is an additional tree at the beginning of the street. So, although mathematical word problems also require verbal skills, such as reading ability, solving such a problem may be easier when students are well able to visualize the respective situation mentally. This way, building a spatial mental model of the problem (e.g., a number line) is beneficial as a problem‐solving strategy in such mathematical tasks.

Further support for this explanation drawing on spatial abilities comes from a study by Wei et al. (2016) on sex differences in estimation in mathematics. Compared to exact calculation in mathematics, estimation (e.g., “What is the best approximate number for 4578 + 5773?”) is believed to rely less on verbal processing but more on spatial representations of number magnitude (Dehaene et al., 1990). Wei et al. (2016) observed that 6th to 8th grade boys and male students outperformed their female peers in a mathematical estimation task, and that this male advantage disappeared when mental rotation skills were controlled for. These findings suggest that the male advantage in some domains in mathematics may be (partly) due to an advantage in visual‐spatial ability.

Further evidence for sex differences in solution strategies comes from a study by Reinert et al. (2017) who compared performance on two number line estimation tasks: a task that required numerical estimation and the application of flexible solution strategies versus a task that allowed for the application of overlearnt strategies. Their findings showed that males had a more pronounced advantage over females when number line estimation required to flexibly apply numerical estimation strategies, compared to when number line estimation allowed for the application of overlearnt strategies.

### Sex differences in mathematical solutions strategies

Although the verbal versus visual‐spatial ability explanation implies that girls are more likely to prefer verbally based solution strategies, whereas boys are more likely to prefer visual‐spatially oriented solution strategies, studies that explicitly investigated sex differences in math strategy use show a reverse pattern (Bailey et al., 2012; Carr & Davis, 2001; Sunde et al., 2020). Several studies showed that boys were more likely to use (verbal) retrieval strategies than girls (Bailey et al., 2012; Carr & Davis, 2001; Sunde et al., 2020), and that they used these strategies faster and more accurately (Bailey et al., 2012; Carr & Alexeev, 2011). Girls, on the other hand, tended to use visual‐spatial strategies such as manipulatives or finger counting (Carr & Davis, 2001) more often and abandoned these strategies more slowly over time than boys (Carr & Alexeev, 2011).

When math problems are more challenging, however, both sexes were found to revert back to using manipulatives, a visual‐spatial strategy (Carr & Alexeev, 2011). This finding is in line with the view that math fact retrieval is a more advanced problem‐solving strategy compared to using manipulatives. This claim is substantiated by evidence that children show a shift from visual‐spatial strategies to verbal strategies as they grow older (De Smedt et al., 2009; Holmes & Adams, 2006). Notably, however, in a study on mathematical word problems, Lowrie and Kay (2001) showed that 6th grade boys and girls were comparably likely to use visual‐spatial solution strategies.

So, findings regarding sex differences in the use of visual‐spatial and verbal solution strategies may also depend on the type of math tasks used. Moreover, findings on mathematical solution strategies are often based on observations of (counting) behaviours and/or self‐reports. Both methods have their limitations. Strategies are not always visible in observable behaviours and the reliability of self‐reports for cognitive processes has been debated (Kirk & Ashcraft, 2001; Smith‐Chant & LeFevre, 2003; Tenison et al., 2014).

### Solution strategies and working memory involvement

The use of visual‐spatial versus verbal strategies during mathematical tasks might be reflected in the contribution of visual‐spatial versus verbal WM in mathematical performance. That is, solving mathematical problems always requires students to understand the content of the problem, translate it into a mental model upon which they then apply their solution strategy, and keep track of processing steps and intermediate results to come to a solution. In other words, mathematical problem solving depends considerably on WM (for a review, see Raghubar et al., 2010), which reflects the ability to temporarily hold and process information (Baddeley, 1986; Conlin et al., 2005).

So, the modality of WM that is relied upon may vary depending on the strategy used. For example, as counting heavily relies on verbal codes this strategy may rely more on verbal than visuospatial WM resources. In contrast, visualization of number lines and dividing sums into smaller parts may rely more on visuospatial WM resources (Cragg et al., 2017). Evidence for such a selective association between strategy use and WM modality comes from research on developmental changes in WM and mathematics. In particular, results from studies during preschool and primary and secondary education indicate that younger children seem to rely more on visual‐spatial WM when learning and applying new mathematical skills, whereas older children seem to rely more on verbal WM after skills have been learned (see e.g., Andersson & Lyxell, 2007; De Smedt et al., 2009; Henry & MacLean, 2003; Kyttälä et al., 2010; Van der Ven et al., 2013). This is possibly due to a shift from visual‐spatial representations and strategies to more verbal representations and strategies (Van de Weijer‐Bergsma, Kroesbergen & Van Luit, 2015). Further evidence for a selective association between strategy use and WM modality comes from research showing that participants’ reported preferred strategy (visual or verbal) is specifically associated with brain activation of areas subserving either verbal or visual‐spatial WM processes (see e.g., Kraemer et al., 2009). This suggests that if girls use more verbal solution strategies in specific math tasks, their verbal WM may be more strongly linked to performance on these tasks. On the other hand, if boys predominantly use visual‐spatial solution strategies in specific math tasks, their visual‐spatial WM may be more strongly linked to performance on these tasks.

Despite the previously mentioned sex differences in verbal and visual‐spatial abilities, however, it should be noted that sex differences in WM ability do not seem to be present yet in childhood (Alloway et al., 2006; Bull et al., 2008; Conklin et al., 2007; Gathercole et al., 2004). A male advantage on visual‐spatial WM tasks only starts to become more apparent during adolescence (for a review, see Voyer et al., 2017), while verbal WM ability seems to stay comparable between sexes, even in adults (Kaufman, 2007; Pauls et al., 2013; Redick et al., 2012; Wang & Carr, 2014).

The finding that sex differences in visual‐spatial and verbal WM do not seem to be apparent during childhood, may be viewed as contradictory to the evidence for a male advantage in visual‐spatial skills and female advantage in verbal skills. However, it might be that, although sex differences in WM are not present (yet) in childhood, prolonged use of preferred visuo‐spatially or verbally based solution strategies may impact the development of the related WM modality and thus explain emerging sex differences in adolescence. Indeed, although interventions specifically aimed at training WM often showed no or only limited transfer effects to WM in daily life or academic contexts (Melby‐Lervåg et al., 2016; Redick et al., 2015), some studies did show the opposite: Higher‐order skills such as WM developed in the context of subject‐matter instruction and practice (e.g. using verbalizations), which in turn supported learning the subject matter (Clements et al., 2001; Mulcahy et al., 2021). As such, sex differences in WM ability may also evolve as a result of prolonged use and experience with certain strategies. Importantly, however, to the best of our knowledge, there are no studies yet that examined possible sex differences *in the relation* between visual‐spatial or verbal WM and (the development of) different mathematical abilities.

Against this background, boys and girls may already be prone to rely more on either visual‐spatial versus verbal solution strategies during mathematical problem solving. Moreover, even when males and females show similar performance on a mathematical task, the solution strategies they used may differ. Sex differences in underlying cognitive processes and strategies earlier in life may therefore underlie the (later) development of sex differences in math achievement. Research on potentially differential contributions of WM resources to the development of mathematical achievement for boys and girls may thus allow for a better understanding of the origins of sex differences in mathematical achievement. As several studies suggested that sex differences in solution strategies (Bailey et al., 2012; Carr & Davis, 2001; Sunde et al., 2020) and math achievement (Filippetti & Richaud, 2016; Geary et al., 2000) may already appear during primary school, this period of development was the focus of the present study.

### The present study

In this study, we investigated sex differences in the contribution of verbal and visual‐spatial WM to the development of math achievement on two different types of math tests: (a) a test requiring mathematical word problem solving and (b) a math fluency test requiring the application of learnt math strategies. We considered data from a large‐scale intervention study on the effects of teacher training in differentiated math education on student math performance.^1^ In the present study, we will examine both the overall level and growth of mathematical achievement over five measurements in two school years and its potentially differential association with visual‐spatial and verbal WM for male and female students. Based on the above elaborations on influences of task characteristics and known sex differences for visual‐spatial and verbal processing between males and females, we pursued the following hypotheses^2^:

1. We expected a male advantage for math word problem‐solving tests as context problems have been argued to be solved more efficiently by strategies that are argued to be more prominent in boys: visual‐spatial and self‐developed solution strategies (e.g., Zhu, 2007). Because word problems also tap into reading ability, and girls tend to show stronger verbal abilities (Hyde, 2016; Levine et al., 2016), the male advantage is expected to be more pronounced after controlling for reading comprehension scores.
2. Our expectations for sex differences on the math fluency test were less consistent: as previous findings indicated that girls may have an advantage in math tasks that primarily require verbal processing and learnt procedures (Wei et al., 2012; Zhu, 2007) one might expect girls to perform better on the fluency test. However, as contrasting findings also indicated that boys tend to use (verbal) retrieval strategies more accurately (Bailey et al., 2012; Carr & Davis, 2001; Sunde et al., 2020), one might also expect boys to perform better on the fluency test.
3. Our expectations regarding sex differences in the association between WM and (development of) achievement on the two math tests are based on the verbal versus visual‐spatial ability explanation (Hyde, 2016; Levine et al., 2016). While we expected girls to show a stronger relation between verbal WM and math achievement, we expected boys to show a stronger relationship between visual‐spatial WM and math achievement.

## METHOD

### Participants

We selected a sample of 3175 children (from 144 classes; 31 schools) from the large‐scale GROW study investigating the effects of a professional teacher development program (Prast et al., 2018). These children were assessed on their math achievement longitudinally over the course of two school years. Children were in grades 2 through 5 during year 1 and in grades 3 through 6 during year 2 of the study. Sample characteristics are presented in Table 1. We used a passive informed consent procedure in which parents received written information about the study and informed the teacher of their child when they did not want their child to participate. The study was approved by the ethical committee of the Social and Behavioral Sciences Faculty, Utrecht University.

**TABLE 1:** Sample characteristics

Grade level in year 1	*n*	% of boys	Age (y;m)	
			*M*	*SD*
Grade 2	811	54.1	7;4	0;5
Grade 3	772	51.3	8;5	0;6
Grade 4	800	48.6	9;5	0;6
Grade 5	792	50.0	10;5	0;6
Total	3175	51.0	8;11	1;3

### Assessment materials

#### Working memory

Children had to complete two online computerized WM tasks suitable for self‐administration in the classroom, the Lion game, and the Monkey game. The Lion game is a visual‐spatial complex span task, in which children search for coloured lions (Van de Weijer‐Bergsma, Kroesbergen, Prast, et al., 2015). In each trial, eight lions of different colours (red, blue, green, yellow, and purple) are presented consecutively for 2000 ms at different locations in a 4 × 4 matrix containing 16 bushes. Children have to remember the last location where a lion of a certain colour (e.g., red) has appeared. After the trial has ended, children use the mouse to click on the correct location. WM load of the task increases from level 1 to level 5 by increasing the number of colours – and hence, the number of locations – children have to remember and update. Each level consists of four items (20 items in total) and no cut‐off rules are applied. We scored the proportion of items recalled correctly. To control for the linear and quadratic effects of age, age‐residualized scores were created by regressing the proportion correct score on age and age‐squared and saving the unstandardized residuals. The Lion game has excellent internal consistency (Cronbach's α between .86 and .90 for different ages), satisfactory test–retest reliability (*ρ* = .71) and good concurrent and predictive validity (cf., Van de Weijer‐Bergsma, Kroesbergen, Prast, et al., 2015).

The Monkey game is a backward verbal span task, in which children are presented with spoken one‐syllable words (Van de Weijer‐Bergsma et al., 2016). Children have to remember the words and recall them in reversed order by clicking on the words presented visually in a 3 × 3 matrix. WM load of the task increases from level 1 to level 5 by increasing the number of words children have to remember (ranging from two to six words). Each level consists of four items (20 items in total) and no cut‐off rules are applied. We scored the proportion of items recalled in the correct order. To control for the linear and quadratic effects of age, age‐residualized scores were created by regressing the proportion correct score on age and age‐squared and saving the unstandardized residuals. The Monkey game has good internal consistency (Cronbach's α between .78 and .89 for different ages), shows good concurrent and predictive validity, and substantial stability over a period of 2 years (*SE* = .52, *p* < .001, after controlling for age *SE* = .41, *p* < .001).

#### Math fluency

The Arithmetic Tempo Test (ATT; De Vos, 1992) is a standardized paper‐and‐pencil test frequently used in Dutch and Flemish education to measure math fluency. The test consists of five sets of 40 addition (+), subtraction (−), multiplication (×), division (÷), and a mixture of the four domains. For each set, children have 1 min to solve as many problems as possible. All problems consist of two‐operand equations with an outcome smaller than 100 and both operands ranging between 0 and 90. The total number of problems answered correctly was used as a dependent variable. Its psychometric properties have been established in a sample of 10,059 Flemish children (Ghesquière & Ruijssenaars, 1994). Correlations between the different measurement occasions in our study showed excellent test–retest reliability (ranging from *ρ *= .85 to .95 between different occasions).

#### Math problem solving

The criterion‐based Cito Mathematics Tests (CMT) are national Dutch tests used to monitor the progress of primary school children (Janssen et al., 2005). These tests primarily consist of contextual math problems. There are two different versions for each grade, one to be administered at the middle of the school year and one at the end of the school year. In each test, five main domains are covered: (a) numbers and number relations, (b) addition and subtraction, (c) multiplication and division, (d) complex math applications, often involving multiple mathematical manipulations, and (e) measuring (e.g., weight and length). From grade 2 through grade 6 several domains are integrated into the math curricula successively: (f) estimation, (g) time, (h) money, (i) proportions, (j) division, and (k) percentages. The items for each domain become more difficult every next grade. Raw test scores are converted to ability scores that increase throughout primary school, enabling the comparison of results of different tests on the same scale (Janssen et al., 2005). Ability scores can vary between 0 (lowest in grade 2) and 169 (highest in grade 6). The reliability for the math tests in different grades ranging from .91 to .97 (Janssen et al., 2005).

There was a significant correlation between total ATT scores and performance on the CITO math problem‐solving test (ranging from *ρ* = .57 to .73 for different measurement occasions).

#### Reading comprehension (Covariate)

The criterion‐based Cito Reading Comprehension Tests (CRCT) are national Dutch tests used to monitor the progress of primary school children in reading comprehension (Feenstra et al., 2010; Weekers et al., 2011). The tests consist of different reading passages, followed by a total of 50 (grades 1 through 4) or 55 (grades 5 and 6) multiple‐choice questions. Raw scores are converted into ability scores that increase throughout primary school, enabling the comparison of results of different versions. Ability scores can vary from −82 (lowest in grade 2) to 147 (highest in grade 6). Reliability was reported as satisfactory (Cronbach α ranges for the different grades from .84 to .93).

### Procedure

Measurements took place on different occasions during two school years (Year 1 and 2; see Table 2 for an overview). Visual‐spatial and verbal WM were assessed using the Lion and Monkey games at the beginning and middle of Year 1. Teachers received an email containing login information for their class and were asked to let all students finish the tasks within a period of 3 weeks.

**TABLE 2:** Measurement occasions

Variable	Year 0	Year 1			Year 2		
	May/June	Sept/Oct	Jan/Febr	May/June	Sept/Oct	Jan/Febr	May/June
Math problem solving	CMT1		CMT2	CMT3		CMT4	CMT5
Math fluency		ATT1	ATT2	ATT3		ATT4	ATT5
Visual‐spatial WM		Lion game					
Verbal WM			Monkey game				
Reading comprehension			CRCT				

Note

Abbreviation: WM, Working memory.

The ATT was administered to small groups of children by the teacher on five occasions during Years 1 and 2. Children in grade 2 completed only the first two ATT scales on addition and subtraction because multiplication is introduced only later during the school year and division is introduced only at the end of grade 2. Children from grade 3 onwards finished all five ATT columns.

The CMT’s were administered in schools as part of their regular progress monitoring program and ability scores were requested from teachers for five different occasions, including one occasion prior to the start of the study (Year 0).

### Data screening

#### Missing values

There were missing data for *n* = 443 (14%) on the Monkey game, and for *n* = 430 (14%) on the Lion game. For the CMT1 to CMT5 scores, data were missing for *n* = 316 (10%), *n* = 220 (7%), *n* = 386 (12%), *n* = 435 (14%), and *n* = 1069 (34%) children, respectively. For the CRCT scores, there were missing data for *n* = 487 (15%) children. For the ATT1 to ATT5 scores, data were missing for *n* = 386 (12%), *n* = 315 (10%), *n* = 186 (6%), *n* = 197 (6%), and *n* = 536 (17%), respectively.

In total, *n* = 1540 (49%) children had complete data on the variables included in the CMT analysis, when reading ability was not controlled for. When reading ability was controlled for this was reduced to *n* = 1357 (43%) children. For the ATT analysis, *n* = 1813 (57%) children had complete data.

The large scale of the study made it unfeasible to keep track of reasons for missing data. However, several reasons can be identified as highly probable. Missing data for the Monkey game, the Lion game, and ATT assessments were most probably due to the absence from school during the time of testing, and in a few cases due to technical issues in the computerized WM assessment. A large number of missing values for CMT5 can be explained by the fact that all students who were in grade 6 in the second year of data collection (*n* = 795) missed data on this particular measurement. This is because many schools administered other tests at the end of grade 6 as part of school policy, to inform tracking decisions with regard to the transfer to secondary school. When CMT (or CRCT) data are missing for children from other grades, this is most likely because children changed schools during the study. In general, we expect that the reasons for missing data were mainly due to the absence of children from school (e.g., due to sickness, dentist visit, and attendance to an official family event). Thus, it would be plausible to assume missing values to occur randomly (Schafer & Graham, 2002).

#### Outliers

In large samples, a few outliers are to be expected (Tabachnick & Fidell, 2007). Four univariate outliers were detected (*Z* scores >3.29) in the ATT1, ATT2, and ATT3 scores, and nine outliers were detected in the CMT2, CMT3, CMT4, and CMT5 scores. In the Monkey game and Lion game respectively, one and seventeen outliers were detected. These outliers were either high or low but realistic scores, and were therefore not removed. Five potential multivariate outliers were detected on the basis of Mahalanobis distances, *χ* ^2^(3) = 16.27. However, the influence of these outliers was negligible (Cook's distance <0.13), and outliers were therefore not removed.

### Data‐analysis

For all analyses, the Mplus statistical package (Muthen & Muthen, 2006) was used. A full estimation maximum likelihood method was applied, because it is robust to non‐normality and can estimate parameters in the case of missing data without imputation.

First, in a two‐level multilevel model, intraclass correlations (ICC) were calculated for all outcome variables to indicate the ratio of the variance between classes to variance within those classes, using grade as a control variable. Following Hox (2002), ICC values of .05, .10, and .15 are considered to be small, medium, and large, respectively. Additionally, a design effect larger than 2 would indicate that clustering in the data needs to be considered for estimation. The design effect was calculated by 1 + (average cluster size – 1) * intraclass correlation (Muthen & Satorra, 1995).

Second, it was examined whether boys and girls differed in their WM and reading comprehension using a multilevel multivariate regression analysis with sex as an independent variable and the Lion game, Monkey game, and CRCT scores as dependent variables. All variables were allowed to covary and were controlled for the grade. Because the aim of this analysis was not to search for the best‐fitting model but to examine the strengths of the relationships between sex and the dependent variables, no fit indices were considered.

**FIGURE 1:**
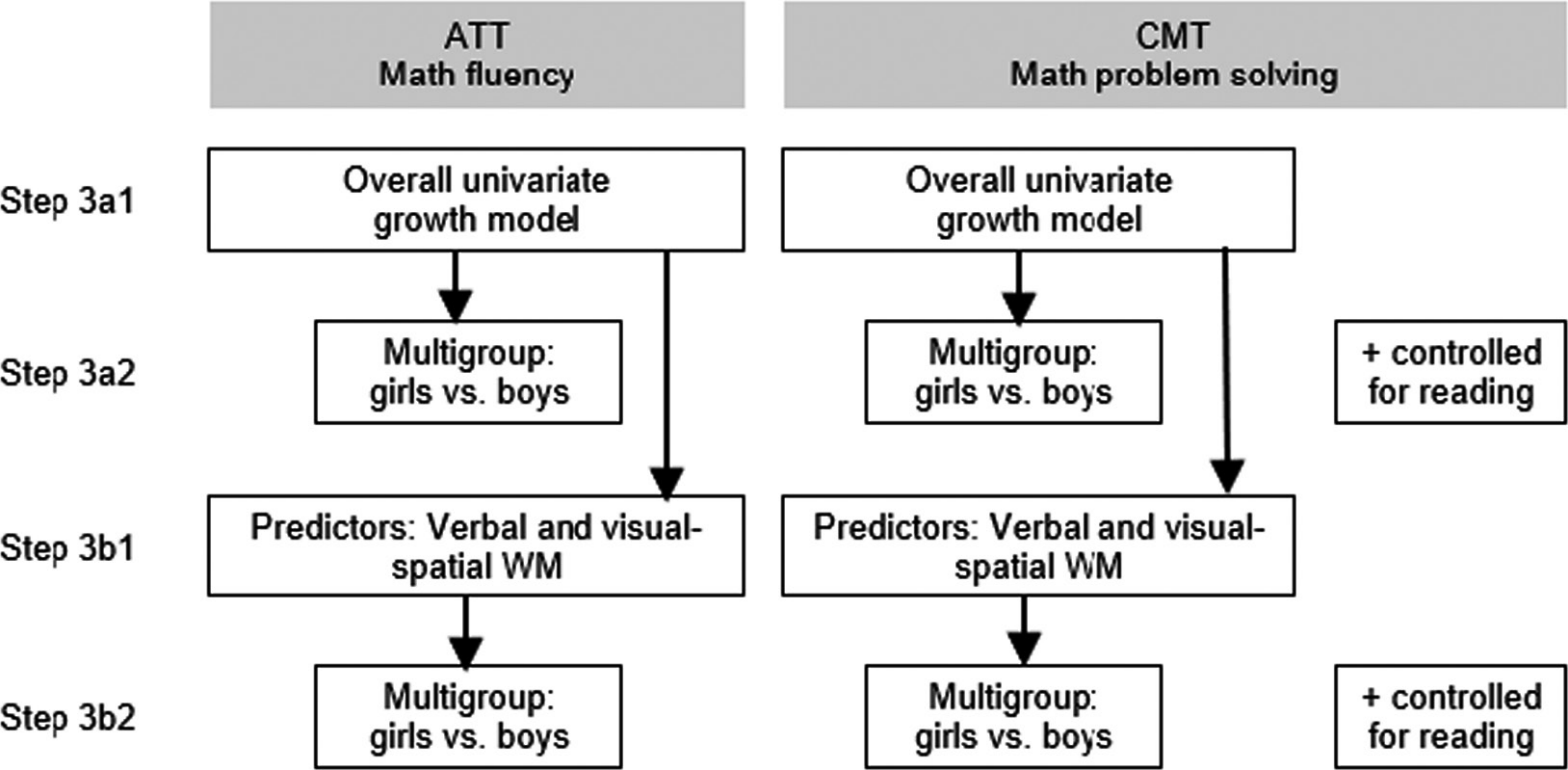
Steps in multilevel multigroup latent growth analysis

Third, we used multigroup multilevel latent growth modelling in several steps (see Figure 1) to examine differences between girls and boys with respect to growth in math performance on the ATT and CMT and the predictive value of WM. In each step, grade level was included as a control variable. Moreover, in each multigroup step, CMT models were run with and without reading ability as a covariate to check whether these suppressed sex differences. ATT models were not run with reading ability as a covariate, since the ATT does not tap into reading comprehension.

In step 3a1, we fitted two overall (i.e., for the whole sample) univariate latent growth curve models, one for ATT performance and one for CMT performance, to investigate the level and growth rate of math achievement. Linear growth was modelled by fixing regressions weights for occasions 1 to 5 at −2, −1, 0, 1, and 2, to prevent collinearity with quadratic slope weights. Additionally, this way of coding better reflects that the intercept does not refer to the initial achievement level but to the overall achievement level instead. Quadratic growth was modelled by estimating a quadratic slope with regression weights for occasions 1–5 fixed at 4, 1, 0, 1, and 4. Model fit indices considered were the comparative fit index (CFI), Tucker–Lewis index (TLI), and root mean square error of approximation (RMSEA). CFI and TLI indicate a good fit of the model to the data when >.95 and acceptable fit when >.90. RMSEA reflects good model fit when <.05 and acceptable when ≤.08 (Browne & Cudeck, 1993). Because of the large sample size, we expected the χ^2^ tests to be significant.

In step 3a2, to examine whether girls and boys differ in math performance levels and growth, we added a within‐level grouping command to estimate parameters (intercept and slope) for girls and boys separately. We allowed intercept and slope means and variances to be freely estimated for girls and boys separately. Additionally, differences between the groups were tested using Wald χ^2^ tests. The CMT model was run with and without reading comprehension as a covariate.

In step 3b1, we extended the two overall models from step 3a1 by considering visual‐spatial and verbal WM as additional predictors (centered around the grand mean and allowed to covary) to test whether individual differences at the level of performance (intercept) and the rate of growth (slope) in math fluency (ATT) and math ability (CMT) were predicted by WM.

In step 3b2, to evaluate sex differences in the predictive value of visual‐spatial and verbal WM for intercept and slope, a within‐level grouping command (i.e., sex) was added again. Dependent differences *within* sex groups in standardized estimates (*SE*s) of visual‐spatial versus verbal WM were tested with Steiger's Z (*Z_H_*) (Hoerger, 2013; Steiger, 1980), taking into account the covariance between the two WM tasks, *SE* = .39 in the ATT model and *SE* .40 in the CMT model, both *p* < .001. Independent differences *between* girls and boys in SEs were tested using a Fisher *r*‐to‐*z* transformation (Lowry, 2013; Steiger, 1980). The CMT model was run with and without reading comprehension as a covariate.

## RESULTS

Descriptive results for all outcome variables are presented in Table 3.

**TABLE 3:** Descriptives, Intra Class Correlations (ICC) and Design Effects for Working Memory (WM), Math Fluency (ATT) and Math Problem Solving (CMT) and Reading Comprehension (CRCT)

Variable	Girls			Boys			Total			ICC	Design effect
	*n*	*M*	*SD*	*n*	*M*	*SD*	*n*	*M*	*SD*		
Visual‐spatial WM	1333	0.68	0.17	1412	0.65	0.18	2745	0.67	0.17	.074	2.51
Verbal WM	1328	0.54	0.15	1404	0.52	0.14	2732	0.53	0.14	.089	2.81
ATT1	1366	16.67	5.27	1423	16.99	5.55	2789	16.84	5.42	.109	3.22
ATT2	1407	18.69	5.33	1453	18.69	5.76	2860	18.70	5.55	.093	2.89
ATT3	1458	19.48	5.51	1531	19.56	5.84	2989	19.52	5.68	.113	3.30
ATT4	1452	20.93	5.84	1526	20.85	6.25	2978	20.89	6.06	.075	2.53
ATT5	1299	21.76	5.67	1341	21.90	6.28	2640	21.83	5.99	.070	2.43
CMT1	1401	69.74	22.12	1458	73.26	22.80	2859	71.54	22.53	.041	1.84
CMT2	1446	77.12	22.45	1509	80.52	22.64	2955	78.86	22.61	.052	2.06
CMT3	1348	82.86	20.74	1441	86.20	20.54	2789	84.59	20.70	.051	2.04
CMT4	1333	89.79	20.24	1407	92.23	19.53	2740	91.04	20.06	.055	2.12
CMT5	1035	90.52	17.26	1071	93.61	16.27	2106	92.09	16.83	.067	2.37
Reading comprehension (CRCT)	1323	31.84	18.04	1366	28.88	18.70	2689	30.33	18.44	.064	2.30

### Intraclass correlations

Table 3 presents ICC’s and design effects for the different variables. ICC’s ranged from small (WM and CMT scores), to medium (most ATT scores), to large (reading comprehension), indicating that the proportion of variance explained by class membership varied between variables. The design effect for almost all variables [1 + (21.38 – 1) *ICC] was greater than 2, indicating that clustering in the data needed to be taken into account during analyses. Although no attempt was made to explain variance at the classroom level, standard errors were corrected for the nested structure using an automatic multilevel modelling setup in all models (Stapleton, 2006). Applying the Mplus mode “type is complex” ensured that part of the model‐variance is attributed to between‐class variance (i.e., variance in achievement outcome existing between classrooms) rather than only to within‐classroom variance.

### Multilevel multivariate regression results

With regard to sex differences in WM and reading ability, the results of the multivariate regression models indicated that, after controlling for grade, girls showed significantly higher scores than boys on verbal WM, *SE* = .17, *p* < .001, as well as visual‐spatial WM, *SE* = .13, *p* < .001, and on reading comprehension, *SE* = .11, *p* < .001.^3^

### Multigroup latent growth curve modelling

#### Overall growth models of math achievement

In step 3a1, the overall quadratic growth model for ATT scores showed acceptable fit, χ^2^(8) = 110.92, *p* < .001, CFI = .983, TLI = .968, RMSEA = .07. Because this model gave a warning and the quadratic slope showed very high collinearity with the linear slope, *SE* = −.90, *p* < .001, a linear growth model was tested, which also provided acceptable fit, χ^2^(13) = 222.60, *p* < .001, CFI = .965, TLI = .960, RMSEA = .07 [90%CI 0.06; 0.08]. This linear model, with a mean intercept factor of 3.46 (*p* < .001) and a mean slope factor of .62 (*p* < .01), was used for further analysis. Individual variation around the intercept factor mean (19.02) and the slope factor mean (0.38) was found to be significant (all *p* < .001), indicating that children differed in their level of math fluency and rate of change over time. Intercept and linear slope showed a significant positive relationship, *SE* = .30, *p* < .001, indicating that children who had a higher overall level of math fluency showed a larger increase. Grade level had a significant positive relationship with the intercept, *SE* = .60, *p* < .001, and the slope, *SE* = .18, *p* < .01, indicating that children in higher grades had a higher overall level of math fluency and showed a larger increase over time.

The quadratic growth model for CMT scores fitted the data very well, χ^2^(8) = 35.28, *p* < .001, CFI = .999, TLI = .998, RMSEA = .03. This model had a mean intercept of 7.46 (*p* < .01), a mean slope of 12.32 (*p* < .001), and a mean negative quadratic factor of −0.27 (*p* = .448). However, since the quadratic factor had a very low value and was not significant, the model was compared to a linear model, which also fitted the data very well, χ^2^(13) = 127.24, *p* < .001, CFI = .995, TLI = .994, RMSEA = .05 [90%CI 0.05; 0.06]. This linear model had a mean intercept factor of 6.43 (*p* < .01), a mean slope factor of 11.66 (*p* < .001). Since a plot of the quadratic and linear model showed that the models were almost identical, further analyses were conducted with the simpler linear model as it facilitates interpretation.

In the linear model individual variation around the intercept factor mean (153.99) and the slope factor mean (2.77) were significant (all *p* < .001), indicating that children differed in their overall level of math problem solving and rate of change over time. The intercept and slope did not covary significantly, *SE* = .01, *p* = .855. Grade level revealed a significant positive relation with the intercept, *SE* = .79, *p* < .000, and a negative relation with the linear slope, *SE* = −.51, *p* < .000, indicating that children in higher grades had a higher overall level of math problem solving but showed a smaller increase over time.

#### Sex differences in math achievement

In step 3a2, including a within‐group level command revealed that intercept parameters for the ATT model differed significantly between girls (3.22) and boys (3.54), *Wald*(1) = 20.19, *p* < .001, indicating that boys showed a higher overall level of math fluency, with a small effect size, Cohen's *d* = .07. The slope parameter showed no significant difference between girls (0.64) and boys (0.61), *Wald*(1) = 1.12, *p* = .291, indicating that they did not differ in their rate of change in math fluency. Covariances between intercept and slope did not differ between girls, *SE* = .32, *p* < .001, and boys, *SE* = .27, *p* < .001, *Z* = −1.5, *p* = .134.

For the CMT model, we found no significant differences in the intercept mean between girls (3.85) and boys (8.14), *Wald*(1) = 0.799, *p* = .372, indicating that girls and boys did not differ in their overall level of math problem solving. The difference in the linear slope mean between girls (11.79) and boys (11.54) was significant, *Wald*(1) = 5.79, *p* < .05, indicating that girls showed a higher rate of change in math problem solving, with small effect size, Cohen's *d* = .15. In this model, covariances between intercept and linear slope were neither significant for girls, *SE* = .04, *p* = .532, nor for boys, *SE* = .03, *p* = .532. Adding reading comprehension as a control variable did not change the findings with regard to differences between girls and boys.

#### Overall predictive value of visual‐spatial and verbal wm for math achievement

In step 3b1, adding visual‐spatial and verbal WM as predictors resulted in acceptable fit indices for the model for math fluency (ATT), χ^2^(19) = 283.91, *p* < .001, CFI = .967, TLI = .957, RMSEA = .07 [90%CI 0.06; 0.07], and good fit indices for the model for math problem solving (CMT), χ^2^(19) = 157.79, *p* < .001, CFI = 1.000, TLI = 1.000, RMSEA = .05 [90%CI 0.04; 0.06]. Intercept means, slope means, and standardized estimates are presented in Table 4. From this table, it can be read that in the overall model for ATT, visual‐spatial and verbal WM explained a significant part of the variance of the intercept, indicating that higher verbal and visual‐spatial WM ability were predictive of higher overall levels of math fluency. Verbal WM was also a significant predictor of the variance in the rate of change, indicating that children with higher verbal WM ability showed stronger growth in math fluency over time. Visual‐spatial WM was no significant predictor of the rate of change.

In the overall CMT model, visual‐spatial and verbal WM also accounted for a significant part of the variance in the intercept, indicating that higher verbal and visual‐spatial WM abilities were predictive for higher overall levels of math problem solving. Visual‐spatial and verbal WM were no significant predictors for the rate of change.

#### Sex differences in predictive value of visual‐spatial and verbal wm for math achievement

In step 3b2, we again performed multi‐group analysis. Intercept means, slope means, and standardized estimates for boys and girls separately are presented in Table 4. To ease the interpretation of findings, path diagrams of the three final models can be found in the Figures [Link], [Link], [Link]. With regard to *math fluency* (ATT) in girls, both visual‐spatial and verbal WM were significant predictors of the overall level of performance. Steiger's *Z* (*Z_H_*) showed that their predictive value did not differ significantly. Verbal WM was a significant predictor of the rate of change in math fluency in girls, while visual‐spatial WM was not, and the difference in predictive value between the two was significant with verbal WM being a significantly stronger predictor of growth in math fluency in girls.

**TABLE 4:** Intercept and Slope Means, Standardized Estimates of Grade, Visual‐Spatial and Verbal Working Memory (WM) to Predict Intercept and Slope Variances in Math Achievement (after controlling for grade), Total Explained Variance (*R* ^2^), and Dependent and Independent Difference between Standardized Estimates (Z_H_ and Fisher *r*‐to‐*z*)

	Math fluency (ATT)				Math problem solving (CMT)				Math problem solving (CMT), controlled for reading			
	Overall model	Girls	Boys	Fisher *r*‐to‐*z*	Overall model	Girls	Boys	Fisher *r*‐to‐*z*	Overall model	Girls	Boys	Fisher *r*‐to‐*z*
Intercept												
Mean	6.25	5.95	6.51		21.36	18.77	24.33		39.16	36.73	42.71	
Grade	.49***	.51***	.48***		.64***	.65***	.64***		.47***	.46***	.46***	
Visual‐spatial WM	.13***	.16***	.11***	1.39	.18***	.21***	.16***	1.4	.14***	.15***	.13***	0.56
Verbal WM	.16***	.14***	.19***	1.41	.24***	.22***	.28***	1.75*	.15***	.13***	.19***	1.69
Reading	NA	NA	NA		NA	NA	NA		.35***	.39***	.34***	
*R^2^*	.41***	.44***	.39***		.73***	.74***	.75***		.79***	.82***	.80***	
*Z_H_*		0.71	2.85**			.029	3.5***			0.67	2.04*	
Linear slope												
Mean	0.73	0.75	0.73		11.55	11.68	11.40		11.35	11.50	11.20	
Grade	.14*	.15*	.14*		−.50***	−.54***	−.47***		−.48***	−.52***	−.44***	
Visual‐spatial WM	.01	.03	−.00	0.82	−.06	−.04	−.08	1.1	−.06	−.06	−.06	0
Verbal WM	.10*	.13*	.07	1.66	.03	.07	−.03	2.74**	.03	.05	.00	1.37
Reading	NA	NA	NA		NA	NA	NA		−.03	−.03	−.08	
*R^2^*	.04*	.06*	.03		.26***	.29***	.26***		.26***	.26***	.27***	
*Z_H_*		3.49**	2.46*			3.11**	1.77			3.65**	2.01*	

Note

Abbreviation: NA, not applicable.

*p < .05; **p < .01; ***p < .001.

In boys, both visual‐spatial and verbal WM were significant predictors of the overall level of math fluency, and Steiger's *Z* (*Z_H_*) showed that verbal WM was a significantly stronger predictor for the level of math fluency than visual‐spatial WM. Although verbal and visual‐spatial WM were both not significant predictors of the rate of change in math fluency in boys, the difference in predictive value was significant: verbal WM was a stronger predictor than visual‐spatial WM.

When sexes were compared, Fisher *r*‐to‐*z* values were not significant, indicating that the predictive value of verbal and visual‐spatial WM for level and rate of change in math fluency did not differ between girls and boys.

With regard to *math problem solving* (CMT) in girls, both visual‐spatial and verbal WM were significant predictors of the overall level of performance, and their predictive value did not differ significantly. However, verbal and visual‐spatial WM were no significant predictors of the rate of change in girls. The difference in predictive value for the rate of change between visual‐spatial WM, which had a small negative influence, and verbal WM, which had a small but positive influence, was significant. Adding reading comprehension as a control variable did not change these findings for girls.

In boys, both visual‐spatial and verbal WM were significant predictors of the overall level of performance, and verbal WM was a significantly stronger predictor for the level of math problem solving than visual‐spatial WM. Verbal and visual‐spatial WM were no significant predictors of the rate of change in boys, and the difference in predictive value was not significant either. Adding reading comprehension as a control variable did not change these findings for boys.

When sexes were compared, Fisher *r*‐to‐*z* values were significant for the predictive value of verbal WM for level and rate of change, but not visual‐spatial WM, indicating that individual differences in verbal WM were more influential in boys than in girls. When reading comprehension was added as a control variable, the difference between boys and girls in the predictive value of verbal WM for overall level and rate of change in math problem solving was no longer significant.

## DISCUSSION

The aim of the current longitudinal study was to evaluate sex differences in (i) the (development of) performance on two types of math tests (i.e., math fluency and math problem solving) and (ii) the potentially differential influence of verbal and visual‐spatial working memory on the (development) of achievement on these math tests for boys and girls. First, we expected a male advantage for contextualized math problem solving, while our expectations for sex differences in math fluency were less pronounced. Second, we hypothesized that because girls show stronger verbal abilities, they would show a stronger relationship between verbal WM and math achievement. Boys, on the other hand, were hypothesized to show a stronger relationship between visual‐spatial WM and math achievement, as boys have stronger visual‐spatial abilities. Our findings indicated that sex differences in math ability were small or completely absent. Furthermore, sex differences in the relation between visual‐spatial/verbal WM and (the development) of math achievement were observed, but not in the expected direction. These findings will be discussed in further detail below.

### Sex differences in math achievement

With regard to sex differences in math achievement, the two math tests showed slightly different patterns, but in general sex differences were only small. With regard to math fluency, we found that boys showed significantly better performance than girls even though with a small effect size. On the other hand, boys and girls did not differ in their rate of development in math fluency over time. As regards math problem solving, boys and girls did not differ significantly in their level of performance. However, girls showed a larger rate of development over time in math problem solving, but again this difference was small. Although these findings contrast our expectations and previous studies showing sex differences in math achievement (Geary et al., 2000), the findings are in line with other studies indicating that sex differences may not (yet) be present in primary school or only minimal (Sewasew et al., 2018; Zhu, 2007). Research by Hutchison et al. (2019) indicates that sex differences in basic numerical processing during childhood are more likely to be an exception than the rule. However, children apply their basic numerical skills in a broader context of math education and math tests, where personal and/or contextual factors (e.g., specific form or content, attitudes, and expectations) may contribute to observed sex differences. Although the type of math test used may be such a factor, our findings indicate that, regardless of the type of math test, sex differences in math achievement seem to be negligible during the primary school years. We must note however, that sex differences have been shown to vary between different math domains (Mullis et al., 2016; OECD, 2018) and with problem difficulty (Bielinski & Davison, 2001; Penner, 2003). Bielinski and Davison (2001), for example, repeatedly replicated an interaction between sex and item difficulty in a study with multiple samples of 4th, 8th, and 12th grade students assessed on different math tests. They observed that boys performed better on more difficult items, while girls perform better on easier items. In this study, sex differences may have been cancelled out as we were not able to differentiate on item difficulty. And although the math problem‐solving test we used taps into various domains of math ability, the available data (total scores) did not allow us to differentiate between different domains. Future studies may examine variations in item difficulty and different subdomains of math problem solving to uncover sex differences in these domains.

Moreover, although sex differences in math achievement may be much less straightforward than assumed earlier, small and subtle initial differences during primary school may become larger over time. Or may appear as a result of situational factors, such as gender stereotypes, and related affective factors, such as math anxiety. Future longitudinal studies should cover the transition between primary and secondary school (and beyond) and may include both personal (e.g., math anxiety and self‐efficacy) and (cross‐cultural) situational factors (e.g., gender stereotypes and instructional design), to be able to examine whether sex differences increase or not, and which factors play a role in whether initial differences are magnified.

### Sex differences in the involvement of verbal and visual‐spatial working memory

The associations between visual‐spatial/verbal WM and math fluency or math problem solving showed some general patterns, both in the overall models and with regard to sex differences. First, both visual‐spatial and verbal WM were significantly associated with math fluency and math problem‐solving performance in general. Associations with WM were stronger for math problem solving than for math fluency, which may be explained by the fact that solving word problems draws on WM abilities more strongly as it requires building a mental model of the problem described – including keeping in mind the respective numbers, ordering them correctly according to the procedure required, and so on. On the other hand, the math fluency test did not require these steps but simply focused on completing basic arithmetic operations. For girls, visual‐spatial and verbal WM turned out to be equally strong predictors of level of performance in both models. For boys, however, verbal WM was observed as a stronger predictor than visual‐spatial WM for the level of performance, even though this was significant for math problem solving only. Differences between sexes were only significant for the association of verbal WM and math problem solving, which suggests that boys relied more strongly on verbal WM when solving word problems than did girls. Although this is in contrast with the verbal versus visual ability explanation for sex differences, these findings are in line with studies that indicate that boys are more inclined to use math fact retrieval (e.g., Bailey et al., 2012; Sunde et al., 2020), which is considered a verbal strategy. Possibly, boys also used this verbal strategy more often when solving mathematical word problems. Notably, the finding that verbal WM was more strongly related to math problem solving in boys than in girls was still present when individual differences in reading ability were taken into account, but no longer reached significance. This indicates that the involvement of verbal WM in *reading* the math problem may have partly accounted for these sex differences, besides the solution strategies employed. One might speculate that demands on verbal WM were higher for students with lower reading ability, leaving fewer WM resources available for the application of verbal solution strategies. This may affect boys more strongly than girls, as they show lower reading ability in general and were more prone to use verbal solution strategies than girls.

Moreover, only verbal (but not visual‐spatial) WM was a significant predictor for the rate of change in math fluency and math problem solving. Here, the pattern of sex differences was more diverse: For girls, those with stronger verbal WM abilities showed a steeper increase in math fluency, but not in math problem solving. For boys, those with stronger verbal WM showed a smaller increase in math problem solving. These findings are not in line with our hypotheses and even contrast our expectation that boys would show a stronger relationship between visual‐spatial WM and math ability, as we argued that they rely more on visual‐spatial strategies. In contrast, these findings suggest that boys rely more on verbal strategies than on visual‐spatial strategies and do this even more so than girls, which is in line with studies on strategy use. According to the study by Carr and Alexeev (2011), for example, boys rely more on fact retrieval than on manipulatives, and at an earlier age than girls.

An implication of our findings may be that boys’ math achievement may benefit more from reading interventions, while girls’ math achievement may benefit more from stimulating automatization of verbal math facts, for example using rote learning. Related to this point we want to stress that the relative strength of an individual in verbal or visual‐spatial WM may determine which strategies they tend to use (Wang & Carr, 2014). It should be noted, however, that it is feasible to assume that individual differences in verbal and visual‐spatial strategies and skills may be larger within groups of girls and within groups of boys than the differences between the sexes.

Affective factors such as math anxiety may also play a role in sex differences in the involvement of WM in math achievement. Research consistently indicated that females seem to be more anxious than males about math (Ganley & Vasilyeva, 2014) and that this difference in math anxiety between sexes is already present in primary and secondary school (Hill et al., 2016). Moreover, a study by Geary et al. (2019) showed that the relation between math anxiety and achievement is stronger in girls, suggesting that they are affected more strongly by the negative effects of math anxiety. This finding is especially relevant when we consider that math anxiety has been suggested to negatively impact performance through its relation with WM (Ganley & Vasilyeva, 2014). It has been argued that intrusive worry‐related thoughts as a symptom of math anxiety consume a significant amount of WM resources, leaving fewer WM resources available for solving the task at hand (Ashcraft & Kirk, 2001). Although evidence on whether visual‐spatial or verbal WM performance is affected most by math anxiety is somewhat mixed, Ganley and Vasilyeva (2014) found that female college students’ visual‐spatial WM functioning was hampered by their heightened worry, and that the resulting sex differences in WM were related to sex differences on a math test. Moreover, math anxiety and related WM depletion may also affect the type of strategies students use (Ramirez et al., 2016). In case math anxiety plays a role in triggering or exacerbating sex differences in math achievement, training math strategies may be an effective way to reduce or prevent a gap between girls and boys. In this context, a study by Passolunghi et al. (2020), for example, showed that math strategy training can improve math achievement and reduce math anxiety.

### Limitations

Besides the limitations we mentioned earlier (i.e., lacking information on item difficulty and specific math domains), our study also has two other important points to consider when interpreting the results. First, WM was measured separately from math achievement and only reflects an indirect relationship (i.e., working memory was not assessed during the performance of a mathematical task). Therefore, further research would be desirable to substantiate our conclusions. For instance, more ‘online’ and direct measures of WM (e.g., using a dual‐task paradigm) and strategies used during math tests may give more specific insights into sex (and individual) differences in verbal versus visual‐spatial strategies applied in math problem solving. Second, it is important to note, that we did not assess the type of strategies used, making it harder to draw a conclusion about how sex differences in WM involvement were related to differences in strategy use between boys and girls. However, collecting information on the specific type of verbal or visual‐spatial strategies used seems important, as different strategies (e.g. verbal counting vs. fact retrieval, or finger counting versus number line estimations) may tap into different WM resources to a different extent. Future studies may further investigate sex differences in strategy use during math and how these relate to WM involvement using self‐report measures on strategy use. Interestingly, brain‐imaging studies indicated that individuals’ self‐reported strategy preferences (verbal vs. visual‐spatial) were significantly associated with activation of the respective brain area's during mental problem‐solving tasks (Alfred & Kraemer, 2017). Experimental designs may also offer other opportunities, such as the design used by Cragg et al. (2017), in which participants were instructed to use specific verbal or visual‐spatial strategies during math problem solving in different conditions, combined with verbal and visual‐spatial dual tasks (to manipulate WM load).

## CONCLUSION

To conclude, although sex differences in math achievement are rather small during primary school, several cognitive and emotional factors may interact to amplify these differences as children grow older. Longitudinal studies taking into account these factors are needed to unravel how (early) childhood experiences affect sex (and individual) differences in mathematical achievement, and the role of problem‐solving strategies and WM. Moreover, interventions targeted at reducing (or preventing) the gap between sexes in math achievement should not focus solely on solution strategies, or reducing math anxiety in girls but should entail a comprehensive approach targeting multiple relevant factors.

## CONFLICTS OF INTEREST

All authors declare no conflict of interest.

## AUTHOR CONTRIBUTION

**Eva van de Weijer‐Bergsma:** Conceptualization; Data curation; Formal analysis; Investigation; Methodology; Project administration; Visualization; Writing – original draft; Writing – review & editing. **Johannes E.H. Van Luit:** Funding acquisition; Supervision; Writing – review & editing. **Korbinian Moeller:** Conceptualization; Writing – review & editing.

## Data Availability

Data available on request from the authors.

## Footnotes

^1^
In an earlier publication (Van de Weijer‐Bergsma, Kroesbergen & Van Luit, 2015), we already examined the influence of visual‐spatial and verbal WM on the level and growth over one school year on performance in the arithmetic math fluency test but not the math problem solving test. Furthermore, analyses were restricted to three measurements of math fluency during one school year. In another publication (Prast et al., 2018), we examined the effect of a teacher training on differentiated mathematics education on student word problem solving. In 19 schools of the current sample, teachers participated in the training program in year 1 (cohort 1) or in year 2 (cohort 2). The remaining 12 schools were in a control condition. Our previous analysis revealed that cohort 1 only showed a lightly larger growth in math achievement during year 1, which was longer observed in year 2. No significant difference between cohort 2 and the control group was found. Therefore, the potential effects of the training on the relation between WM and match achievement can be considered negligible.

^2^
Hypotheses were formulated based on the literature and before conducting the analyses. Hypothesis 2 is more exploratory as the literature points towards two possible directions.

^3^
Standardized estimates in these analyses are only standardized for the outcome measure and therefore reflect how many standard deviations girls score higher than boys.
