# Supplementary figures and images for: Sex differences in the association of math achievement with visual‐spatial and verbal working memory: Does the type of math test matter?
Source: Br J Psychol. 2022 Mar 29;113(3):798–819. doi: 10.1111/bjop.12562 (PMC9544364; doi:10.1111/bjop.12562)

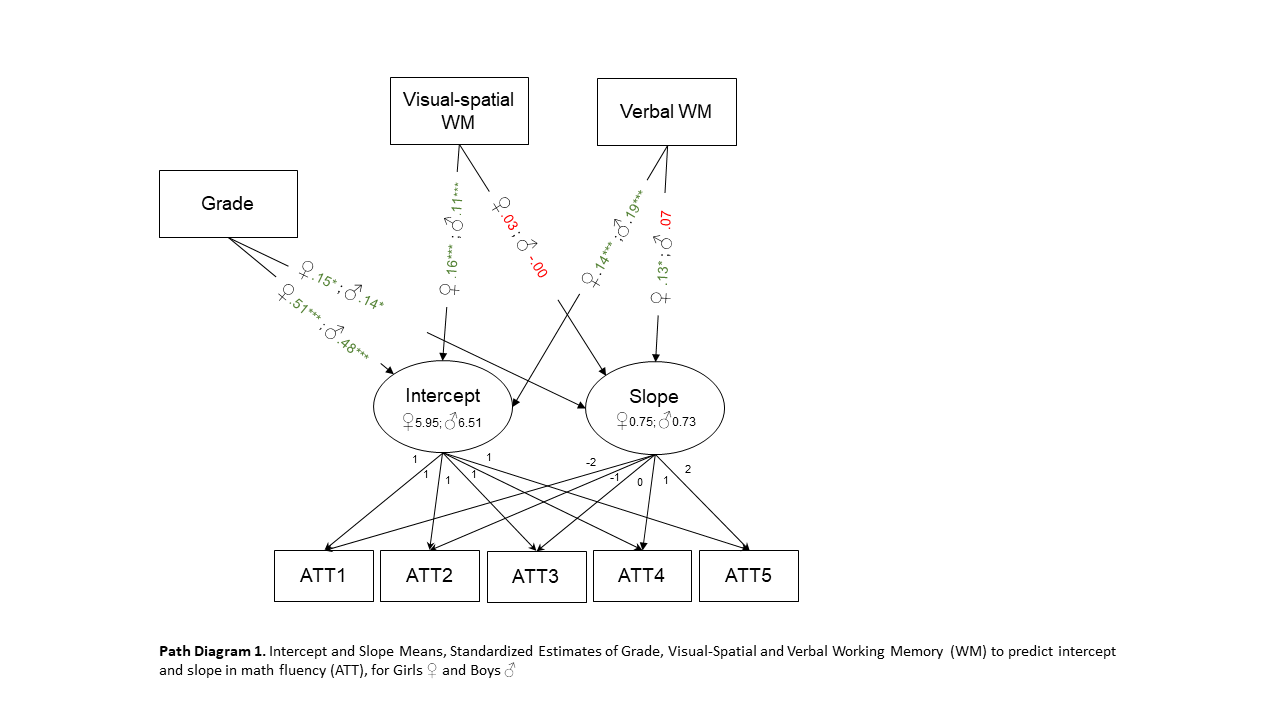

Supplement: Supplementary file 1 [file BJOP-113-798-s001.TIF]

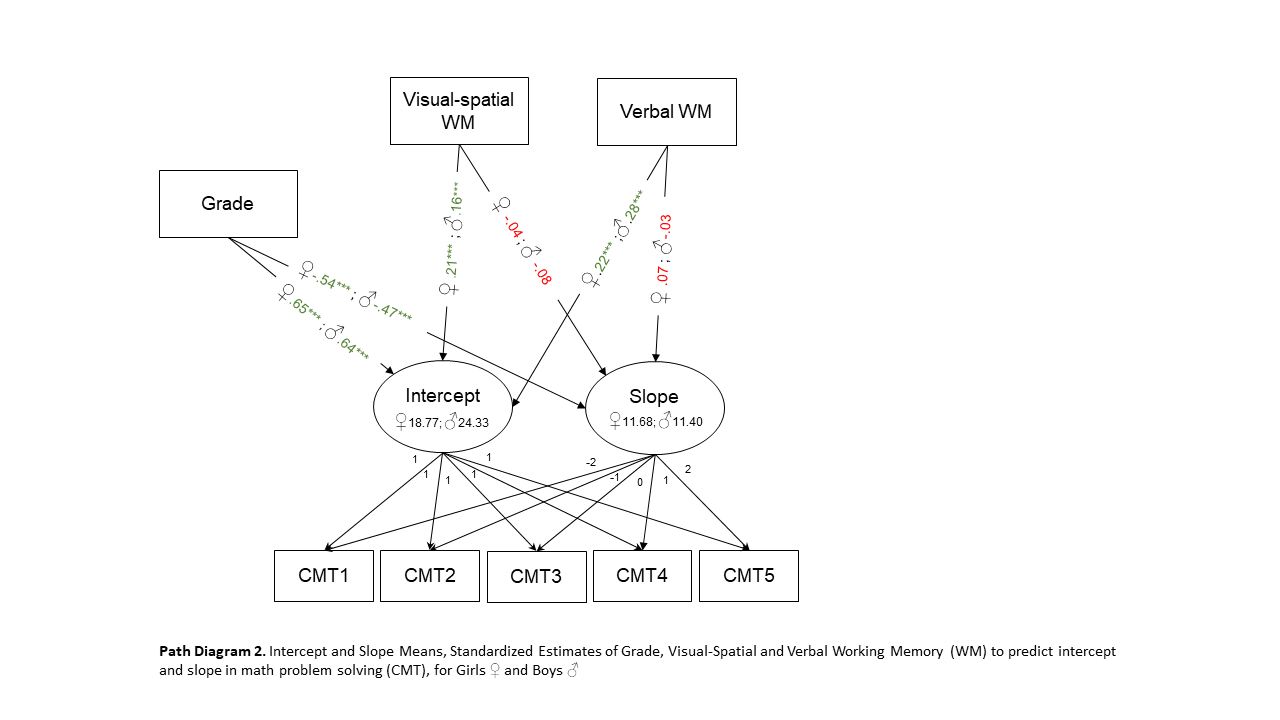

Supplement: Supplementary file 2 [file BJOP-113-798-s003.TIF]

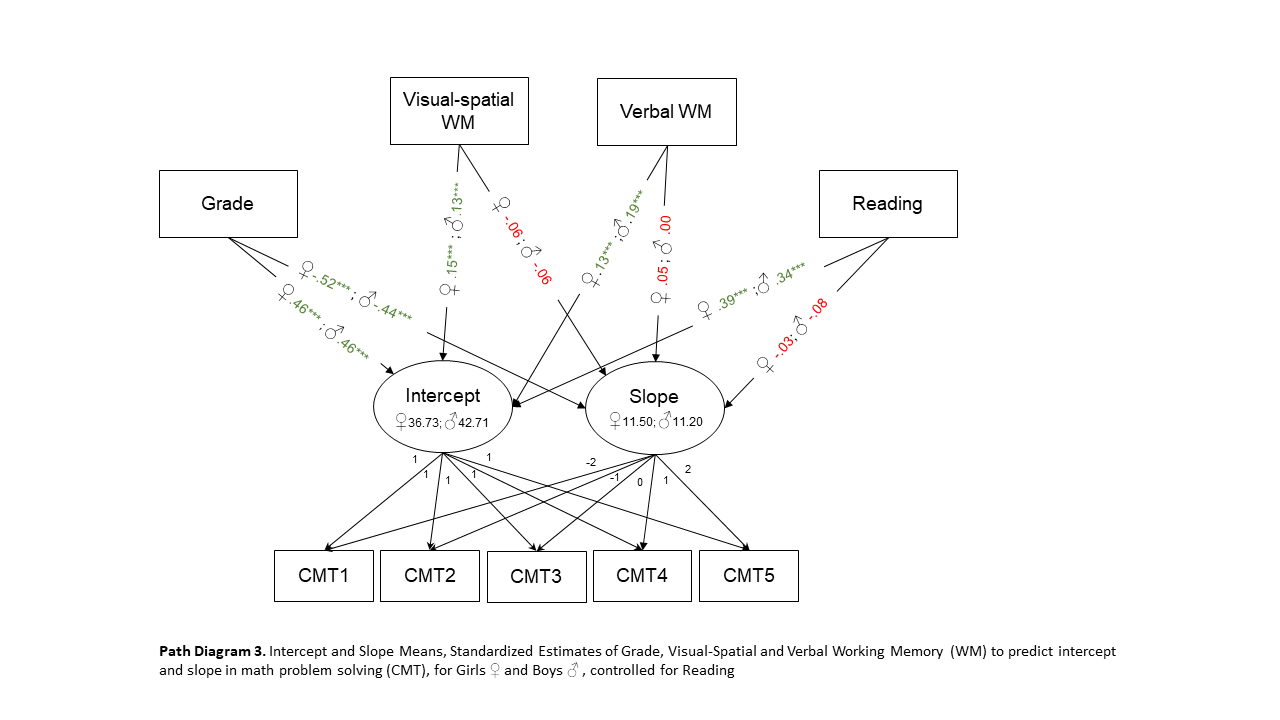

Supplement: Supplementary file 3 [file BJOP-113-798-s002.TIF]
